# The heat of longevity: sex differences in lifespan and body temperature

**DOI:** 10.3389/fphar.2024.1512526

**Published:** 2024-11-25

**Authors:** Chiara Ruocco, Maurizio Ragni, Enzo Nisoli

**Affiliations:** Center of Study and Research on Obesity, Department of Medical Technologies and Translational Medicine, University of Milan, Milan, Italy

**Keywords:** dietary restriction, lifespan, genetic variability, sex differences, thermoregulation, mitochondrial biogenesis, ageing markers, age-related disease

## Abstract

Dietary restriction (DR) has long been recognized as a powerful intervention for extending lifespan and improving metabolic health across species. In laboratory animals, DR—typically a 30%–40% reduction in caloric intake—delays aging and enhances mitochondrial function, oxidative defense, and anti-inflammatory pathways. In humans, findings from the CALERIE™ trial confirm DR’s potential benefits, with a 25% caloric reduction over 2 years resulting in reduced visceral fat, improved cardiometabolic health, and favorable gene expression changes linked to proteostasis, DNA repair, and inflammation. However, recent research in genetically diverse mouse populations reveals that the impact of DR on lifespan is substantially modulated by genetic background, underscoring the importance of individual variability. Additionally, emerging evidence challenges previous assumptions that lower body temperature universally benefits lifespan extension, with data indicating complex relationships between thermoregulation, sex, and longevity. These findings underscore the need for nuanced approaches to DR in both research and potential therapeutic applications, with considerations for genetic and sex-specific factors to maximize healthspan and lifespan outcomes.

## Introduction

Laboratory mice and rats are typically maintained on *ad libitum* (AL) feeding regimen, allowing free access to food and water. In contrast, dietary restriction (DR)—a reduction of caloric intake by 30%–40% from the average AL intake starting around sexual maturity—has been shown to significantly extend lifespan, making it a widely recognized geroprotective intervention (Green et al., 2022). Since McCay et al. (1935) first reported DR’s lifespan-extending effects in rats, DR has become a standard model for longevity research, emphasizing reduced caloric intake rather than nutrient restriction. While restricting single nutrients alone does not reproduce the lifespan extension of DR, limiting proteins, branched-chain amino acids, or specific other amino acids (e.g., methionine, isoleucine, threonine, or tryptophan) without reducing total calories has independently shown positive effects on lifespan in rodents [reviewed by Mihaylova et al. (2023)].

Despite DR’s efficacy, adherence to such regimens remains challenging, prompting investigations into the physiological and molecular mechanisms mediating its effects. Proposed mechanisms include enhanced mitochondrial biogenesis (Nisoli et al., 2005), improved oxidative stress defenses, adaptive anti-inflammatory responses, and reduced cellular senescence, although a complete mechanistic picture remains elusive (Green et al., 2022). A complicating factor in DR studies is the substantial difference in feeding patterns between DR and AL groups: DR-fed mice consume their daily ration within ∼2 h, leading to prolonged fasting phases (Acosta-Rodríguez et al., 2017). Recent findings indicate that this prolonged fasting itself is critical for DR’s metabolic and geroprotective effects, as similar benefits can be achieved with a prolonged fasting period alone, even without calorie reduction (Pak et al., 2021). This distinct feeding behavior, however, complicates comparisons between DR- and AL-fed animals, as metabolic and physiological responses vary with fasting duration. Further research by Pak and colleagues reveals that DR-associated improvements in insulin sensitivity, adiposity, metabolite profiles, and tissue-specific regulation of mechanistic target of rapamycin 1 (mTORC1) activity are heavily influenced by fasting duration and tissue context, rather than by DR alone (Pak et al., 2024). Thus, fasting duration appears critical to understanding DR’s physiological impacts, suggesting that temporal factors (e.g., time-restricted feeding) may be as crucial as caloric reduction in DR regimens.

Recent studies suggest that DR may also attenuate cellular senescence markers in humans (Aversa et al., 2024). The Comprehensive Assessment of Long-term Effects of Reducing Intake of Energy (CALERIE™) trial has demonstrated that a 25% reduction in caloric intake over 2 years in young and middle-aged individuals without obesity led to sustained weight loss, reduced visceral fat, with modest muscle loss, and improvements in cardiometabolic health and blood pressure, all without compromising quality of life (Shen et al., 2021; Kraus et al., 2019). Through linear mixed-effect models, researchers observed DR-associated shifts in gene expression related to proteostasis, circadian rhythm, DNA repair, mitochondrial function, apoptosis, and inflammation (Das et al., 2023). Collectively, these findings indicate that DR may help reduce age-related risk factors also in humans.

## Genetics matters more than diet for lifespan

In a recent issue of Nature, Di Francesco and colleagues investigated the effects of DR and intermittent fasting (IF) on health and lifespan using a genetically diverse cohort of 960 female mice (Di Francesco et al., 2024). The study explored two levels of DR (20% and 40%) and IF (1 or 2 days of fasting per week). While all interventions extended lifespan, only DR increased the mortality doubling time, and the ability to maintain body weight during handling emerged as the strongest predictor of lifespan. Other predictors included changes in immune cells, red blood cell distribution width, and retention of adipose tissue later in life. Lifespan was heritable (h^2^ = 0.24), with genetic background having a greater influence than dietary interventions. Previous research has highlighted physiological adaptations to DR in both rodents and humans—such as improved glucose homeostasis, lower energy expenditure, decreased body temperature, and preserved metabolic flexibility—as potential mechanisms for lifespan extension (Redman and Ravussin, 2011; Guijas et al., 2020). However, Di Francesco and colleagues found no significant associations between lifespan and fasting glucose, energy expenditure, or metabolic flexibility. Surprisingly, higher body temperature, contrary to expectations, was moderately linked to increased lifespan. These findings suggest that while dietary restriction induces significant metabolic changes, their relevance to lifespan extension may be limited, indicating the need to explore alternative biomarkers of aging.

The exclusive use of female mice in this study raises critical questions about the generalizability of the findings. Although it is common practice to use female mice to avoid the aggressive behavior often observed in cohoused male mice, especially in resource-limited experimental groups like those undergoing DR (Behnke and Sewell, 1994), this approach neglects potential sex-specific responses to dietary interventions. Sexual dimorphism is a well-known factor influencing lifespan and metabolic traits, with numerous studies reporting distinct physiological responses between males and females (Austad and Fischer, 2016). For instance, females generally have a longer lifespan than males across species, and hormonal differences, particularly the protective effects of estrogen, play a significant role in modulating metabolic pathways and stress resilience (Austad, 2006; Kane et al., 2018). Studies have also shown that DR and IF can elicit divergent effects on males and females, including differences in body weight regulation, fat distribution, and insulin sensitivity (Redman and Ravussin, 2011).

More specifically, Sanchez-Alavez et al. (2011) demonstrated that differences in core body temperature (Tc) contribute to the sex-specific longevity observed in C57BL76J mice. In young females, Tc was influenced by the estrous cycle but was overall higher than in males; this difference became more pronounced in older mice, where age eliminated the estrous-related variations. Interestingly, while Tc homeostasis is centrally regulated by the sexually dimorphic hypothalamic preoptic area, these differences were dependent on the gonads. These results may explain, at least in part, the linkage observed by Di Francesco et al. (2024) between higher body temperature and increased lifespan, challenging the previous observations that body temperature is beneficial for lifespan extension (Conti et al., 2006; Zhao et al., 2022).

It highlights that the reliance on single-sex cohorts—whether male or female—limits the ability to fully understand the biological complexity underlying responses to dietary restriction and related interventions. Incorporating both sexes to capture the influence of sexual dimorphism on lifespan and healthspan outcomes will improve the translational relevance of these findings to human populations, where sex differences in aging are well documented.

## Conclusion

This commentary underscores the complexity of DR and its interplay with genetic and sex-specific factors in shaping lifespan. Di Francesco et al. (2024) study highlights that while DR extends lifespan, genetic diversity exerts an even greater influence. Their findings on the role of body temperature challenge conventional views, suggesting that higher, rather than lower, body temperature could be advantageous for lifespan. Moving forward, incorporating both sexes in future DR studies is essential to capture the full scope of biological variability in lifespan and healthspan, with significant implications for translational studies targeting human aging. Additionally, this work emphasizes that temporal aspects—such as fasting duration—must be considered in DR regimens, as they may prove as impactful as calorie reduction itself. These insights pave the way for more personalized dietary interventions that account for individual genetic and biological variability, optimizing healthspan and potentially longevity.

## Data Availability

The original contributions presented in the study are included in the article/supplementary material, further inquiries can be directed to the corresponding author.
